# Three-layered ventricular septum of the helical heart: functional anatomy and clinical relevance

**DOI:** 10.1186/1749-8090-8-S1-O106

**Published:** 2013-09-11

**Authors:** MJ Kocica, M Ristic, D Cvetkovic, Lj Soskic, E Nestorovic, AF Corno, VI Kanjuh, V Lackovic

**Affiliations:** 1 Clinic for Cardiac Surgery, UC Clinical Centre of Serbia, Belgrade, Serbia; 2 Alder Hey Royal Children Hospital, Liverpool, UK; 3 Serbian Academy of Science and Arts, Belgrade, Serbia; 4 Institute for Histology and Embriology, Medical School UC Belgrade, Belgrade, Serbia

## Background

Helical ventricular myocardial band (HVMB) is global three-dimensional anatomical model which defines principal, cumulative vectors, integrating tissue architecture and net forces developed within the ventricular mass. Objective of this study is to demonstrate functional macroscopic anatomy of the interventricular septum (IVS) and to emphasize its clinical relevance.

## Methods

Three bovine and two porcine hearts ware prepared according to Torrent-Guasp’s technique. Special dissection technique was undertaken with non- toothed forceps, scalpel and scissors. Blunt dissection, using finger peeling technique was applied to identify predominant direction of the linear (fiber) and laminar (layer) fiber pathways within the myocardial mass.

## Results

IVS displays significant fiber disarray at the boundaries of LV and RV free walls, and contains an intriguing structure that may be freshly examined by the HVMB dissection. These dissections contradict the concept that the interventricular septum belongs to the LV, since both ventricles participate in its formation. Ascending and descending segments od the HVMB provide the origin and significance of mayor “septal fiber crossing”. Conventional low resolution ultrasound imaging of the ventricular septum previously identified the border of this crossing as hyper-echogenic “septal line”. We suspect the overlap of the crossing of descending and ascending segments creates this “bright line”. Histological analyses have shown that septal RV and LV fibers create a connective tissue true space. Coronary artery septal branches run through this space, a fact, which may be useful in Ross’ procedure.

## Conclusion

IVS belongs to both ventricles. Anatomical dissections of the IVS and boundaries of LV and RV free walls have demonstrated significant changes in principal fiber orientation, explaining IVS three-layered functional anatomy. The functional significance of this septal fiber organization has to be further examined.

